# Comparative Study of Ferroelectric and Piezoelectric Properties of BNT-BKT-BT Ceramics near the Phase Transition Zone

**DOI:** 10.3390/ma11030361

**Published:** 2018-03-01

**Authors:** David Andres Fernandez-Benavides, Aixa Ibeth Gutierrez-Perez, Angelica Maria Benitez-Castro, Maria Teresa Ayala-Ayala, Barbara Moreno-Murguia, Juan Muñoz-Saldaña

**Affiliations:** Centro de Investigación y de Estudios Avanzados del IPN, Lib. Norponiente No. 2000, Fracc. Real de Juriquilla 76230, Querétaro, Qro. Mexico; davidfernandez@cinvestav.mx (D.A.F.-B.); aigutierrez@cinvestav.mx (A.I.G.-P.); angelica.benitez@cinvestav.mx (A.M.B.-C.); maria.ayala@cinvestav.mx (M.T.A.-A.); barbara.moreno@cinvestav.mx (B.M.-M.)

**Keywords:** lead-free ceramics, BNT/BKT, sintering, morphotropic phase boundary, functional properties

## Abstract

We report a comprehensive comparative study of ferroelectric and piezoelectric properties of BNT-BKT-BT ceramics through the MPB (morphotropic phase boundary) zone, from the rhombohedral to the tetragonal phases in the system (97.5−*x*)(Bi_0.5_Na_0.5_)TiO_3_ + *x*(Bi_0.5_K_0.5_)TiO_3_ + 2.5(BaTiO_3_), where *x* = 0 to 24.5 mol %. The structural transitions were studied by XRD patterns and Raman spectra. The MPB was confirmed between *x* = 10 and 12.5 mol % BKT. The dielectric/ferroelectric/piezoelectric properties of the BNT-BKT-BT system are maximized in the MPB region exhibiting a dielectric constant of 1506, a remanent polarization of 34.4 μC/cm^2^, a coercive field = 36.9 kV/cm, and piezoelectric values of *d*_33_ = 109 pC/N, *k_t_* = 0.52, and *k_p_* = 0.24. Changes in microstructure as a function of BKT content are also presented and discussed.

## 1. Introduction

Ferroelectric and piezoelectric ceramics are commonly used in several applications such as FE-RAMS, actuators, sensors, bio-sensors, transducers, and energy conversion devices, among others [1,2,3]. Lead zirconate titanate (Pb(Zr,Ti)O_3_ (PZT) is one of the most studied and used ferroelectric materials due to its remarkable performance [1,4]. However, toxicity concerns of PZT are particularly due to the high PbO vapor pressure during sintering steps [5,6], as well as its instability when used or disposed in aqueous environments [7]. In the last decade, big efforts have been made to find alternative materials, which are well summarized in several review papers [8,9,10,11,12,13]. Apart from the well-known barium titanate BaTiO_3_ (BT), three lead-free perovskites are considered the principal candidates to replace PZT, namely: (a) bismuth-based, bismuth-sodium titanate (Bi_0.5_Na_0.5_)TiO_3_ (BNT) and bismuth-potassium titanate (Bi_0.5_K_0.5_)TiO_3_ (BKT); (b) potassium sodium niobates, (K,Na)NbO_3_ (KNN); and (c) barium calcium zirconate titanates (Ba,Ca)(Zr,Ti)O_3_ (BCZT) [3,12].

Bismuth-based ceramics have been extensively explored in the last decade and BNT has been particularly recognized as an important lead-free ferro/piezoelectric material with rhombohedral symmetry (*R*3*c*) [14,15]. It has been reported that BNT exhibits strong ferroelectric properties, such as a large remanent polarization (*P_r_*) of 38 μC/cm^2^ and a high coercive field (*E_c_*) of 73 kV/cm synthesized by a solid state reaction of mixed oxides and sintering at 1000 °C [15,16]. Recent reports based on high-resolution X-ray diffraction and Rietveld refinement have, however, pointed out BNT evidence of lower symmetry at room temperature attributed to a monoclinic phase with a *Cc* space group [17,18]. Particularities in processing BNT might be leading to these differences. However, a broad range of properties have been reported, which can be associated with conductivity issues representing a problem in its poling process and thus in its piezoelectric performance [10,12,19].

On its side, bismuth-potassium titanate (Bi_0.5_K_0.5_)TiO_3_ (BKT), with tetragonal crystal structure (*P*4*mm*) [20], shows densification difficulties during pressureless sintering, and has been preferably obtained by hot pressing leading to a *P_r_* = 27.6 μC/cm^2^ and a *E_c_* = 53 kV/cm [21].

Finally, barium titanate, BaTiO_3_ (BT) with a tetragonal crystal structure (*P*4*mm*) [15], is the most widely studied ferroelectric material. BT shows piezoelectric and dielectric constants at room temperature of 400 pC/N [22] and 3750 (fine grained ceramic, pressureless sintered) [23], respectively, and exhibits a relatively low Curie temperature [11].

A preferred processing route of Bi-based perovskites in bulk is through the synthesis from mixed oxides and carbonates as precursors by a conventional solid-state reaction. This route allows the modification of the BNT properties by the formation of solid solutions with BKT and BT, among others [10,24]. The solid solutions are formed due to the substitution of different A- or B-site cations in the perovskite type structure (*ABO*_3_), causing distortions in the unit cell. A morphotropic phase boundary (MPB) is formed by varying the *K^+^* or *Ba*^2*+*^ cation content but maintaining the BNT-rich composition, where the individual mentioned crystalline phases coexist and give rise to enhanced ferro/piezoelectric properties compared to unmodified BNT ceramics [11,25,26]. Furthermore, the reported ferroelectric and piezoelectric properties from the ternary system BNT-BKT-BT within the MPB zone are already comparable with PZT [9,26]. An MPB zone has been reported in the (100−*x*) BNT+ *x* BT system, in which the composition has been claimed to correspond to *x* = 6 to 8 mol % BT [19]. Another example is the (100−*x*) BNT + *x* BKT system, where the MPB range definition has been reported by different authors in a wide range of compositions from *x* = 12 to 25 mol % BKT, exhibiting a clear discrepancy [27,28,29,30,31,32].

In the BNT-BKT system, fine grained microstructures and a reduction in the coercive field from 63 to 36 kV/cm are obtained as the BKT content increases from 0 to 10 mol % [33]. The reported combination of processing parameters, specially based on the milling of mixed oxides and a solid-state reaction seeking for optimization as a function of composition, e.g., in the MPB zone, are frequently contradictory leading to the formation of spurious phases, a fine-grained microstructure, a poor density, structural defects, and thus poor ferroelectric and piezoelectric behavior. In several cases, the reported ferro/piezoelectric behavior does not correspond to a ceramic with an MPB composition [15,24,25,29,34]. Again, the precise composition of the MPB in the BNT-BKT-BT ternary system is still a topic in debate since different composition ranges have been reported.

On the other hand, studies focusing on the variation of chemical composition going from the rhombohedral to the tetragonal structure through the MPB in the BNT-BKT-BT system have been also reported elsewhere [15,27,35,36,37], but there is a lack of data related to a comprehensive analysis of the structural characteristics of the samples in this system and their link to ferro/piezoelectric behavior.

This work seeks to include the different aspects of the effect of composition on the ferroelectric and piezoelectric properties of BNT-BKT-BT ceramics, in order to confirm the range of compositions for phase transitions from tetragonal to rhombohedral. The structure, microstructure, dielectric properties of the samples in the different compositions, and their effect on the ferroelectric and piezoelectric behavior are presented and discussed in this article.

## 2. Materials and Methods

### 2.1. Sample Preparation

Samples in compositions (97.5−*x*) (Bi_0.5_Na_0.5_)TiO_3_ + *x*(Bi_0.5_K_0.5_)TiO_3_ + 2.5(BaTiO_3_) with *x* = 0, 3, 6, 9, 10, 12.5, 20, and 24.5 mol %, were prepared by mixed powder oxides using the following raw materials: Bi_2_O_3_, TiO_2_, BaCO_3_ (Sigma Aldrich, St. Louis, MO, USA), Na_2_CO_3_ (Meyer, CDMX, Mexico), and K_2_CO_3_-1.5H_2_O (J.T. Baker, J. T. Baker Chemical, Phillipsburg, NJ, USA), all of them having purities over 99%. These precursors were milled for 2 h in a planetary mill (Retsch, Haan, Germany) with 30 mL of methanol and Y_2_O_3_ -stabilized zirconia balls as grinding media based on a 10:1 ball to powder ratio in weight percent. The powder mixtures were then dried at 100 °C for 24 h and subsequently three times calcined at 920 °C for 5 h with a heating rate of 5 °C/min to complete the solid-state reaction. Thereafter, the calcined powder was milled using an SPEX 8000D mixer mill system (SPEX SamplePrep, Metuchen, NJ, USA) and sieved with a 44 μm mesh to avoid particle agglomerates. Disk-shaped green samples were uniaxially pressed with 3.4 MPa using a hardened steel die of 9.5 mm in diameter. These samples were then pressureless sintered in a chamber furnace (Thermolyne, Thermo fischer Scientific, Waltham, MA, USA) at 1120 °C for 5 h with a heating rate of 5 °C/min. Ag-electrodes were painted on both faces of the buttons to perform dielectric, ferroelectric, and piezoelectric measurements.

### 2.2. Sample Characterization

#### 2.2.1. Density, Structural, and Microstructural Characteristics

The theoretical density for each composition was calculated by the law of mixtures, considering the density of the pure phases of (Bi_0.5_Na_0.5_)TiO_3_, (Bi_0.5_K_0.5_)TiO_3_, and BaTiO_3_, which were 5.99 g/cm^3^ (ISCD 98-006-3231), 5.94 g/cm^3^ (ISCD 98-007-7833), and 6.15 g/cm^3^ (ISCD 98-004-6093), respectively. The density was determined by the Archimedes method using a precision balance (Mettler Toledo AB204-S, Bern, Switzerland).

Structural characteristics of the samples were measured by X-ray diffraction in a Rigaku Dmax 2100 diffractometer (Rigaku Corp, Tokyo, Japan), with CuKα radiation (*λ* = 1.5406 Å) and a step size of 0.02°. The structural characterization was complemented by Raman spectroscopy with a confocal micro-Raman spectrometer (LabRam HR Evolution, Horiba Jobin-Yvon, Villeneuve d'Ascq, France) carried out at room temperature.

Characterization of the microstructure of sintered ceramics was performed by scanning electron microscopy (SEM) using an electron microprobe analyzer (EPMA JXA-8530F, JEOL, Akishima, Japan) at a 6 kV electron acceleration voltage and a secondary electron detector (FESEM JEOL 7610F (Tokyo, Japan).

Thermal etching was performed on polished samples in order to reveal the microstructure. Further determination of grain size was done through image analysis by Feret’s method taking 210 grains per composition from several micrographs. Grain size distributions were fit to lognormal functions using Origin software (OriginLab Corp., Northampton, MA, USA). A Kolmogorov-Smirnov test with 95 percent of confidence was performed to validate log-normality for all distributions.

#### 2.2.2. Dielectric, Ferroelectric, and Piezoelectric Properties

The capacitance at 1 kHz and the permittivity of the samples were obtained from impedance spectroscopy measurements using an impedance analyzer (Keysight E4990A, Santa Rosa, CA, USA) based on the following equations:

(1) $$\varepsilon^{\prime }=\frac{C\times d}{A\times \varepsilon_{0}}$$

where, *C* is the measured capacitance, *d* the thickness, *ε*_0_ the permittivity of free space, and *A* the area of the capacitor.

The remanent polarization, *P_r_* and coercive field *E_c_* were both determined from *P*-*E* hysteresis loops obtained with a ferroelectric tester (Radiant Precision LC system, Radiant Technologies, ABQ, NM, USA).

Samples were then poled at room temperature applying an electric field of 60 kV/cm by using a VDC source at 3 kV for 1 h at a frequency of 1 Hz in a bath of silicone oil to prevent the dielectric breakdown. Thereafter, the piezoelectric coefficient (*d*_33_*)*, capacitance (*C*_0_), and dissipation factor (*tanδ*) were measured with a piezometer device (Piezotest PM300, Tanjong Pagar, Singapore). The minimum impedance (*Z_min_*), resonance (*f_a_*)—antiresonance (*f_r_*) frequencies and phases were obtained using the impedance analyzer. Starting from the obtained values, the mechanical (*Q_m_*) and electrical (*Q_e_*) quality factors, and electromechanical coupling factors (*k_t_* and *k_p_*) were calculated by the following equations:

(2) $$Q_{m}=\frac{1}{4\pi C_{0}Z_{min}\Delta f}$$

(3) $$Q_{e}=\frac{1}{tan\delta }$$

(4) $$k_{t}=\sqrt{\frac{\pi f_{r}}{2f_{a}}tan\left(\frac{\pi }{2}\frac{\left(f_{a}-f_{r}\right)}{f_{a}}\right)}$$

(5) $$k_{p}=\sqrt{\frac{2.54\left(f_{a}-f_{r}\right)}{f_{r}}-{\left(\frac{\left(f_{a}-f_{r}\right)}{f_{r}}\right)}^{2}}$$

The densification, dielectric, ferroelectric, and piezoelectric behavior of the samples were analyzed to compare the effects of the BKT-compositions from the rhombohedral to the tetragonal zone through the MPB.

## 3. Results and Discussion

### 3.1. Structural Analysis

#### 3.1.1. Characteristics of Samples by X-ray Diffraction

**Figure 1:**
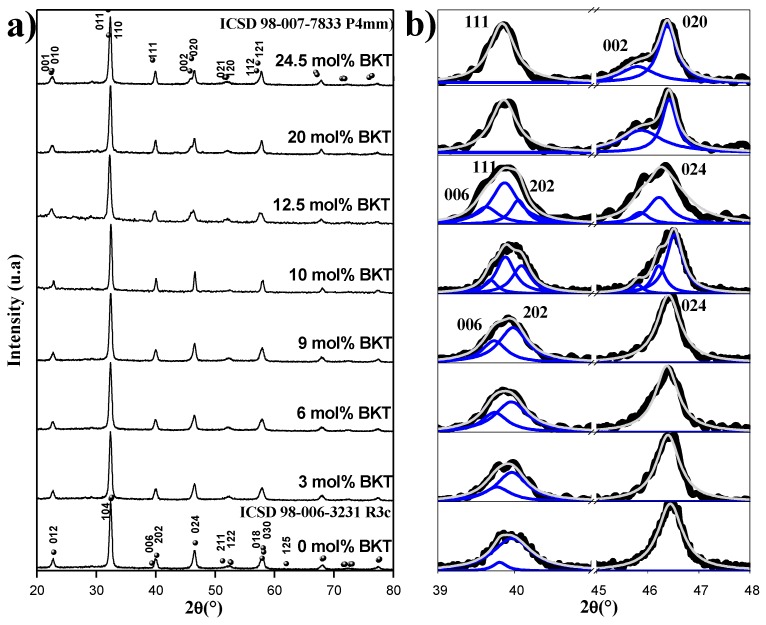
XRD patterns of sintered ceramics in compositions (97.5−*x*)BNT + *x*BKT + 2.5BT with *x* *=* 0, 3, 6, 9, 10, 12.5, 20, and 24.5 mol % BKT (**a**) from 20–80 in 2θ(°) and (**b**) deconvoluted peaks between 39–48 in 2θ(°).

The ferroelectric and piezoelectric behavior of the BNT-BKT-BT samples directly depends on the quality of the ceramic, in terms of a full formation of the substitutional solid-state solution, the homogeneity of chemical composition, and thus long-range ordering conditions. The correspondent diffraction patterns of the *x* = 0 to 24.3 mol % BKT samples are shown in Figure 1a, where a correlation with the characteristic peaks of a perovskite-type crystal structure is shown with no evidence of secondary phases. The transition from rhombohedral to tetragonal symmetry as the BKT content increases is more evident in Figure 1b. The samples with *x* = 0 to 9 mol % BKT show the (006) and (202) characteristic peaks, in the range of 38 to 42°, and (024) between 44 and 48° in 2-theta of the rhombohedral symmetry (*Rc*3). In the samples with *x* = 20 and 24.3 mol % BKT, the splitting of the planes (002) and (200) and the single peak of the plane (111) from 38 to 42° in 2-theta corresponding to a tetragonal symmetry (*P*4*mm*) can be observed. In some cases, the XRD patterns show broadening in specific ranges, which implies the coexistence of more than one phase. As mentioned before, a deconvolution procedure was undertaken for all XRD spectra. This was performed by using the Origin software and a Lorentzian function. The fitting was achieved with a *χ*^2^ under 0.5 and *R*^2^ over 0.96 for all compositions. The experimental diffraction patterns and deconvoluted peaks are all shown in Figure 1b.

The XRD patterns of *x* = 0 to 9 mol % BKT samples were successfully fitted, identifying peaks that match with a rhombohedral symmetry. For the samples with *x* = 20 and 24.5 mol % BKT, the expected fit with tetragonal peaks was successfully achieved. In both cases, the deconvoluted peaks show coherence, not only for the compositions, but also for the expected theoretical relative intensities. Finally, for the samples with *x* = 10 and 12.5 mol % BKT, a six-peaks fit was performed on the experimental diffraction patterns, corresponding to the total expected planes for both rhombohedral and tetragonal phases in a morphotropic composition.

#### 3.1.2. Raman Spectroscopy

**Figure 2:**
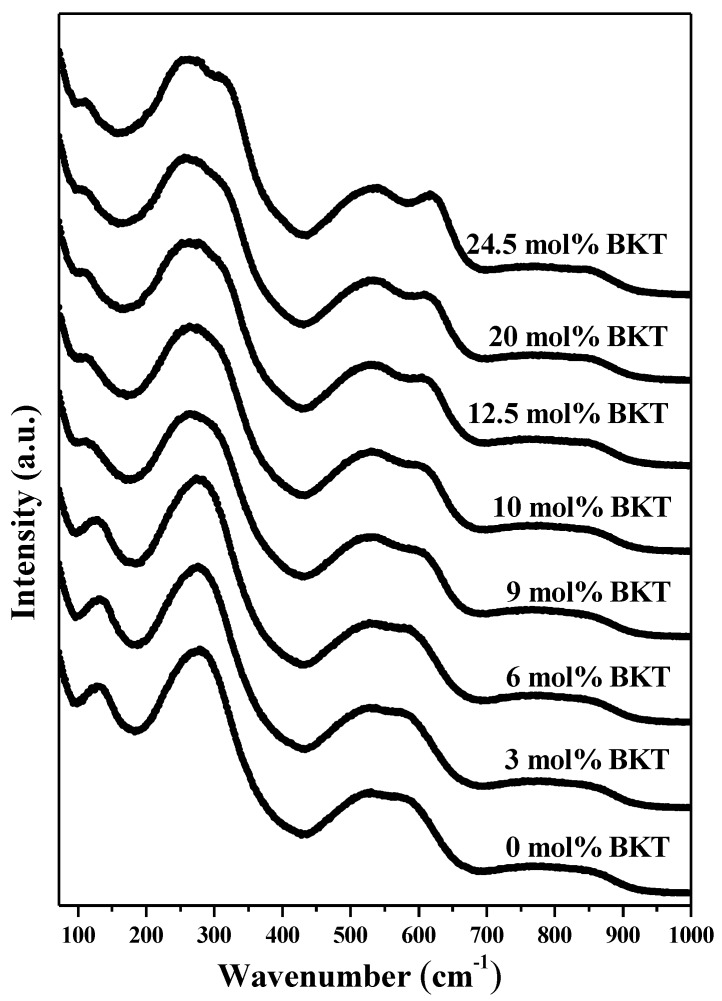
Raman spectra recorded in a wavenumber between 80 to 1000 cm^−1^ of sintered ceramics in compositions (97.5−*x*)BNT + *x*BKT + 2.5BT with *x* = 0/3/6/9/10/12.5/20/24.5 mol % BKT.

The crystal structure and the physical properties of these materials can be analyzed by changes in phonons behavior of the Raman spectra identified as changes in the frequency or intensity of the peaks, which explain the phase transition in the structure of the samples [38]. Figure 2 shows the Raman spectra of *x* = 0 to 24.5 mol % BKT samples in a wavenumber range from 80 to 1000 cm^−1^. The obtained spectra show similar characteristics consisting of overlapped broad bands, which are mainly attributed to the lattice disorder in the A-site due to the substitution with four heterovalent cations, in this case *Bi*^3*+*^, *Na^+^*, *K^+^*, *Ba*^2*+*^ in the perovskite structure [39,40,41].

**Figure 3:**
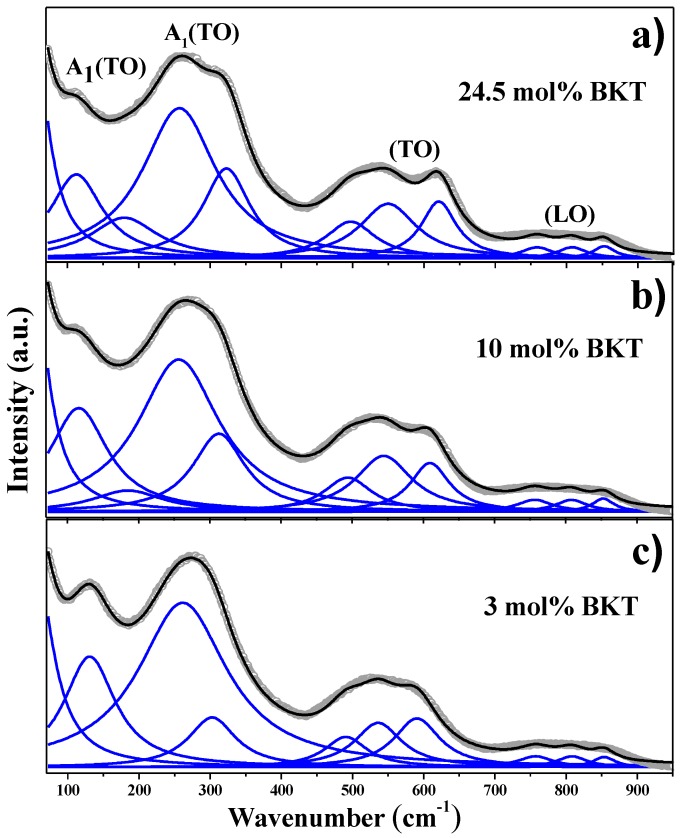
Deconvolution of Raman spectra from sintered ceramics with (**a**) 24.5 mol % BKT; (**b**) 10 mol % BKT; and (**c**) 3 mol % BKT. The gray and dark lines correspond to the experimental and deconvoluted curves, respectively.

Figure 3 shows the Raman spectra deconvoluted with FITYK^®^ using a Lorentzian function. The fitting was achieved with a *χ*^2^ under 0.058 and *R*^2^ over 0.99 for all compositions. In the Raman spectra of the samples with *x* = 0 to 6 mol % BKT, nine peaks were successfully deconvoluted (Figure 3c), whereas in the samples with *x* = 9 to 24.5 mol % BKT, the fitting was only achieved with ten peaks (Figure 3a,b). This methodology is similar to that reported by Kreisel et al. [39] for a BNT-BKT system.

**Figure 4:**
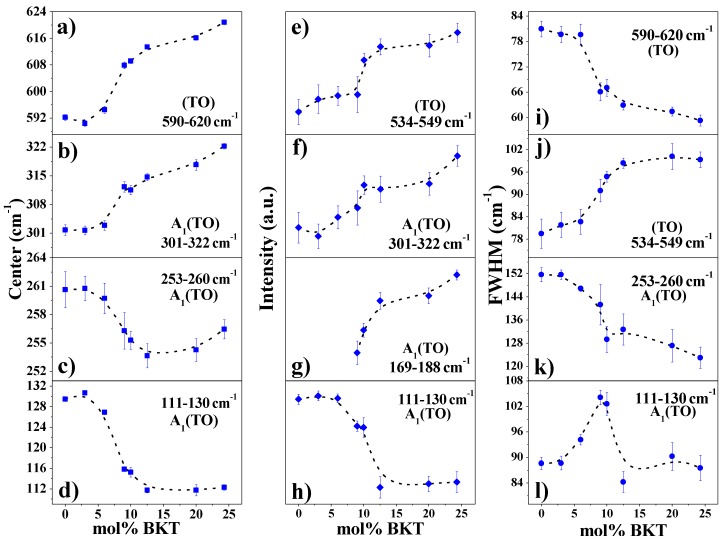
Variations in (**a**–**d**) center, (**e**–**h**) intensity, and (**i**–**l**) FWHM of the Raman spectra deconvoluted peaks from studied compositions as a function of mol % BKT content.

The peak centered between 111 and 130 cm^−1^ has been assigned to *A*_1_*(TO*_1_*)* mode, which has been associated with the A site vibrations: Na-O, K-O, and Ba-O [39,40], and is sensitive to phase changes in the crystal structure of the perovskite [38]. Further increase in substitutional *K*^+^ ions in A sites leads to a decrease in intensity and shifts towards smaller wavenumbers (Figure 4d,g) due to a mass increase. On the other hand, at lower wavenumbers (<70 cm^−1^), Bi-O vibrations are expected since the mass of Bi is higher compared to the other cations.

The band with the highest intensity is observed between 253 and 260 cm^−1^ and has A_1_ symmetry, which corresponds to Ti-O vibrations. It is well known that changes in frequency of this mode are due to the tilting of the *TiO*_6_ octahedron [38,42]. The splitting of this mode into three bands has been previously reported elsewhere and associated with a tetragonal phase [41]. Moreover, the Raman modes in the ranges between 301–322 and 190–200 cm^−1^ have been consistently related to the presence of tetragonal phase [39,41,43]. The former shifts toward higher wavenumbers and its intensity increases with the BKT content (Figure 4b,f). The latter appears first in samples with compositions between *x* = 9 and 24.5 mol % BKT, but in the range of 169–188 cm^−1^ (Figure 4g). The reason of this shifting can be associated with the variations in composition from the current BNT-BKT-BT ternary compared to the reported range, in BNT-BT or BNT-BKT systems [39,40].

Finally, the bands located at frequencies in the range of 534–549 and 590–620 cm^−1^ (Figure 4a,e,i,j) are associated with the *TiO*_6_ octahedron vibration mode [38,40], and are dominated by vibrations that mainly involve oxygen displacements. These are sensitive to changes in composition and their behavior while *K*^+^ increases, leading to clear evidence of structural changes.

The observed changes in frequency, intensity, and FWHM of some Raman modes of the studied compositions, mainly those located at 111–130, 301–332, 534–549, and 590–620 cm^−1^, show clear tendencies that describe the structural transition from a rhombohedral to a tetragonal phase as a consequence of the increase of mol % BKT. The aforementioned behavior is fully consistent with the results from the XRD analysis.

In general, the observed characteristics are in good agreement with previous reports of BNT [38], and BNT-based binary ceramic systems such as BNT-BT [40,41,43], BNT-BKT [39,44], and BNT-BZT [45].

### 3.2. Microstructure

**Figure 5:**
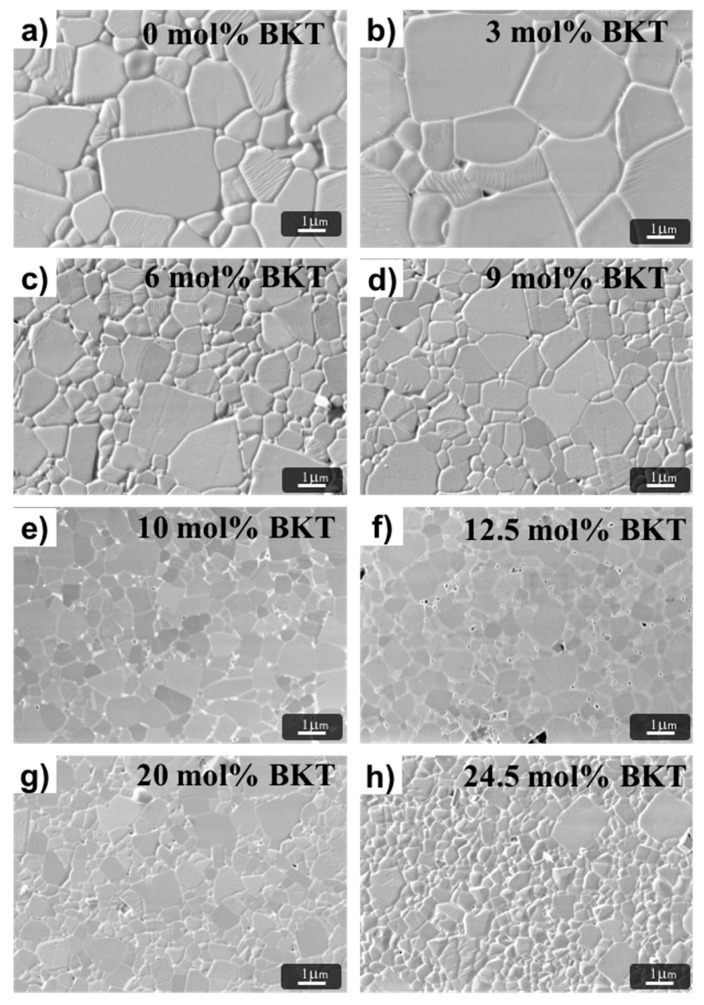
SEM micrographs of polished and thermally etched sintered ceramics in compositions (97.5−*x*)BNT + *x*BKT + 2.5BT with *x* *=* 0, 3, 6, 9, 10, 12.5, 20, and 24.5 mol % BKT for (**a**–**h**), respectiely.

All sintered samples exhibit densifications in the range of 95.1–97.1% of the theoretical density. Typical SEM micrographs of the prepared ceramics are shown in Figure 5. From these, a microstructure with equiaxially shaped grains can be observed. Low porosity is an important quality factor in order to maximize ferroelectric or piezoelectric properties, since it directly affects the electromechanical coupling factor (*k_p_)* [46].

The microstructure analysis showed a lognormally grain size distribution. As can be observed in Table 1, average grain size first slightly increases before decreasing monotonically from the 3 mol % BKT sample as a function of BKT content, leading to a fine-grained microstructure. A similar behavior has been reported in BNT-BKT and BNT-BKT-BT systems [28,30,32,47].

However, in the compositions well inside the tetragonal phase, parallel to the decreasing tendency in grain size with the BKT content, grains with larger sizes remain visible in the microstructure. Interestingly, in the compositions corresponding to the MPB (10–12.5 mol % BKT), the grain size distribution becomes homogeneous.

### 3.3. Electromechanical Analysis

#### 3.3.1. Dielectric Constant

**Figure 6:**
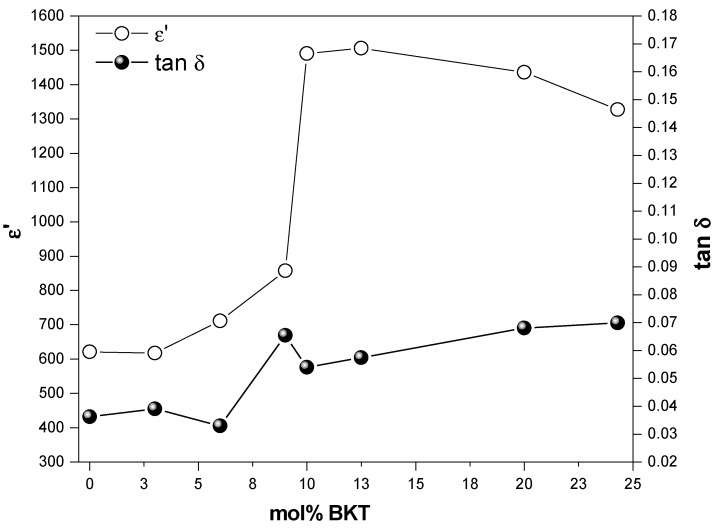
Dielectric properties of the prepared samples measured at 1 kHz and room temperature as a function of mol % BKT.

Considering that the capacitance measurements were done in a quasi-static regime at 1 kHz [48], the relative dielectric permittivity (ε*) and dielectric loss (*tanδ*) were obtained and plotted as a function of BKT content (Figure 6). The phase difference between dipole reorientation and applied electric field gives rise to an energy dissipation effect, which is known as dielectric relaxation, and it can be studied from the frequency-dependent dielectric function behavior:

(6) $$\varepsilon^{*}\left(\omega \right)=\varepsilon^{\prime }\left(\omega \right)-i\varepsilon^{\prime \prime }\left(\omega \right)$$

where, *ω =* 2*πf* is the angular frequency and *f* is the oscillatory field frequency (expressed in Hz). This complex function is composed of a real part *ε*′ (dielectric constant, in phase with the applied electric field) and an imaginary part *ε*″ (dielectric losses, related to absorption or energy loss phenomena). Loss factor *tanδ* is defined as the dissipation and absorption energy ratio (*ε*′*/ε*″).

Additions of BKT in molar compositions from 0 to 12.5 mol % BKT show a continuous increase in dielectric constant, leading to an average value for the relative dielectric permittivity of 650 for *x* < 9 mol % BKT. Maximum values of 1490.5 and 1505.8 are observed in samples with 10 and 12.5 mol % BKT, respectively, which are within the morphotropic zone reported elsewhere [15,36]. These observations are consistent with early reports, which emphasize the improvement of the dielectric and ferro/piezoelectric properties of compositions belonging to MPB of the ternary system [49,50].

It is worth mentioning that the dielectric constant decreases when BKT content is higher than 12.5 mol %, which corresponds to the compositions with tetragonal symmetry. For this range, the grain-size distribution shows a fine-grained microstructure, for which the relative dielectric permittivity is found to be gradually decreasing from 1505.8 to 1327.4 for 12.5 to 24.5 mol % BKT, respectively. This behavior is fully consistent with the increasing tendency of the loss factor (*tan δ*) and is in good agreement with classical experimental and theoretical reports about the electrical, elastic, dielectric, ferroelectric, and piezoelectric behavior of well-characterized ferroelectric ceramics, such as BT and PZT [23,51,52,53,54,55].

The *tanδ* oscillates from 3 to 4% for compositions between 0 to 6 mol % BKT, which are relatively low values and tend to increase between 5 and 7% for 9 mol % and higher BKT contents. Thus, BKT in low proportions shows low values of both the dielectric losses and the permittivity of the material.

The coupling between the dielectric constant with the microstructure has been understood from the analysis of the internal stresses near the grain boundaries as a function of the increase of tetragonality degree in the rhombohedral-tetragonal phase transition of several ferroelectric systems [55].

#### 3.3.2. Ferroelectric Hysteresis

**Figure 7:**
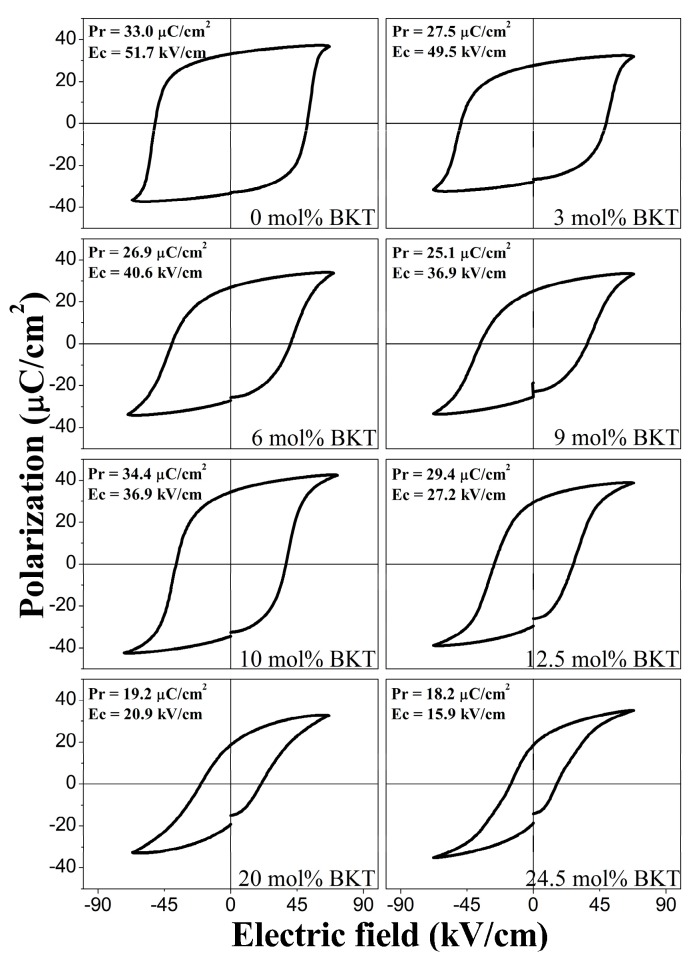
Polarization (*P*)—electric field (*E*) hysteresis loops measured at 1 Hz and room temperature as a function of composition.

The *P*-*E* hysteresis loops measured at electric fields of 6 kV/mm are shown in Figure 7. All curves evidence a typical ferroelectric behavior and saturated non-conductive *P*-*E* loops. Both the *P_r_* and *E_c_* exhibit clear tendencies as the BKT content increases. The remanent polarization and the coercive field in the sample with 0 mol % BKT is 33 μC/cm^2^ and 51.7 kV/cm, respectively, with the latter being the highest one among the samples. When increasing the BKT content, *E_c_* decreases monotonically to a minimum value of 15.9 kV/cm in the sample with 24.5 mol % BKT, whereas *P_r_*, apart from the morphotropic composition (10–12.5 mol % BKT), tends to decrease to a value of 18.2 μC/cm^2^.

It has been reported that the BNT phase shows a *P_r_* of 38 μC/cm^2^ and a *E_c_* of 73 kV/cm [15], which are similar to the ones obtained for the current sample prepared with 0% BKT. The effect of BKT additions on the *P_r_* and *E_c_* values of the BNT-BKT-BT system has been reported in different ways. For instance, in the BNT-BKT binary system, similar tendencies with increasing BKT content are reported, consistently showing that the *P_r_* values reach a maximum in the MPB composition and *E_c_* decreases with BKT additions [30,56]. As mentioned before, the MPB region in the BNT-BKT binary system has been reported by different authors to be located inside a wide range of compositions between 12 and 25 mol % BKT [27,28,29,30,31,32]. Since our system has a fixed low amount of BT and considering our processing conditions, comparisons with BNT-BKT systems are pertinent to identify where, in the discrepancy of MPB compositions, we are located.

In some of these works, one fact that is fully consistent with our results is in compositions with high BKT contents, beyond the MPB region, *P_r_* and *E_c_* values tend to decrease monotonically. Again, these results are fully in accordance with the hysteresis loops of the ternary system for the samples prepared with 20 and 24.5 mol % BKT. In a similar way, Wang et al. reported that additions of BKT up to 20 mol % decrease the coercive field in a BNT-BT system in the MPB composition [35].

The current reported values of *P_r_* for the MPB (10 mol % BKT) in the BNT-BKT-BT show maximum values for *P_r_* (34.4 C/cm^2^), while *E_c_* is still decreasing until it reaches a value of 27.2 kV/cm. These results show similar values and behavior when compared to Yueming et al. [29], where the highest value of *P_r_* (31.4 C/cm^2^) is observed in a composition around 12 mol % BKT with a decreasing tendency of *E_c_*.

On the other hand, *P_r_* tends to decrease, where the crystalline phase determines the characteristics of the *P*-*E* loops [23,48,52,53,54]. The MPB has been defined as a compositional phase boundary at which the two adjacent phases in a phase diagram have equal Gibbs free energy [57]. In the BNT-BKT-BT system, the morphotropic phase boundary region is still under discussion in the literature [37].

According to this, the structural analysis of the samples in this study (Figure 1) shows a phase transition from rhombohedral to tetragonal symmetry around the compositions with 10 and 12.5 mol % BKT (87.5 mol % BNT-10 mol % BKT-2.5 mol % BT to 85 mol % BNT-12.5 mol % BKT-2.5 mol % BT). Again, the structural features of the MPB region in the BNT-BKT-BT system are still a topic of debate and it is expected that by defining the coexistence of two phases, the increased polarizability with an applied electric field is related to the coupling of the energy states of tetragonal and rhombohedral phases, allowing enhanced domain reorientation and thus poling of the ferroelectric ceramic. That is to say, eight directions of polarization of the rhombohedral (111) and six of the tetragonal (100) symmetry are considered in the MPB range of compositions, and higher polarization values are expected [30].

From the current results, we confirm that the compositions inside the MPB tend to enhance the ferroelectric properties in the BNT-BKT-BT system, which is the case of the samples prepared with 10 and 12 mol % BKT. This finding is consistent with different reports claiming that the adequate incorporation of BKT should maximize the ferroelectric and piezoelectric properties [25,35].

The observed behavior of the *P_r_* can also be explained with changes in the microstructure, specifically in the grain size, as shown in Figure 5. Variations in grain size also directly affect the permittivity and later the ferroelectric properties of the material. It is well known that the domain width scales with the grain size [53]. In a fine-grained microstructure, the number of grain boundaries contributes with additional pinning points inhibiting domain switching and leading to a decrease of ferroelectric properties. For larger grains, the domain walls have more mobility that results in a complete orientation of the polarization vectors [54].

#### 3.3.3. Piezoelectric Characteristics

Table 2 summarizes the dielectric, ferroelectric, and piezoelectric behavior of the prepared samples. From these data, it can be easily confirmed that the permittivity (*ε’*) increases proportionally with the BKT content, reaching its highest value in compositions with 10 and 12.5 mol % BKT and revealing characteristics of the morphotropic range according to previous reports [15,36,49,50].

The *k_p_* and *k_t_* in the samples reach maximum values of 0.24 and 0.52, respectively, in the MPB range. On the other hand, the off-resonance piezoelectric constant (*d*_33_) also exhibits a value of 126 pC/N in the MPB.

Regarding the piezoelectric properties, apart from *d*_33_ values, the rest of the measurements are all similar to those reported in the literature. For instance, the mechanical quality and electromechanical coupling factors are comparable with previous reports [15,35,36]. Even though the *d*_33_ values are slightly lower, they show a clear tendency with the BKT content.

Finally, it is notorious that the transition phase zone is mainly defined by the composition, but can change for specific processing conditions. For instance, for Bi-based lead free ferroelectrics, such as BNT and BKT, the presumable volatilization of *Bi*^3*+*^, *Na^+^*, and *K^+^* ions during calcination or sintering steps directly impacts the intrinsic polarization [44,58,59,60]. As a result, the formation of non-stoichiometry spurious phases leads to a resistivity decrease [12,58,59]. In the same way, differences in diffusion rates from A-site cations due to the intrinsic properties of *Bi*^3*+*^, *Na^+^*, *K^+^*, or *Ba*^2*+*^ cations contribute to heterogeneities in stoichiometry in the bulk. Experiments in diffusion couple tests have demonstrated the high diffusion rates of Bi_2_O_3_ compared to other oxides, such as Fe_2_O_3_ [61]. Similar behavior cannot be neglected in literature reported Bi-based perovskites and of course not in the current samples.

More recently, a crossover effect from relaxor type to ferroelectric behavior has been observed with prolonged sintering times for Bi-based perovskites. For instance, in BKT ceramics, a spontaneous relaxor-ferroelectric phase transition occurs in coarse-grained ceramics after sintering for 30 h or more. These results are evidenced in a dielectric permittivity curve as a function of temperature at different frequencies [62,63]. According to this, the disorder degree in the BNT-BKT-BT system naturally determines the ferroelectric behavior since it should be affecting the dipole-dipole interactions in a straightforward manner.

However, a systematic study of the effect of processing parameters on the aforementioned properties still needs to be done. The use of experimental methodologies such as mixtures design and simultaneous optimization, should lead to a further understanding of the relationship between compositions and properties of the BNT-BKT-BT ternary system.

## 4. Conclusions

The MPB range was confirmed between 87.5 BNT-10 BKT-2.5 BT and 85 BNT-12.5 BKT-2.5 BT samples after analyzing a number of compositions by XRD and Raman as a function of BKT content. A heterogeneous grain size distribution was observed in all samples and a monotonically decrease as a function of the BKT content. However, a microstructure with homogeneous grain size was observed in the samples with MPB compositions.

The MPB samples show maximum values in different properties such as a dielectric constant of 1506, a remanent polarization of 34.4 μC/cm^2^, and piezoelectric values of *d*_33_ = 109 pC/N, *k_t_*= 0.52, and *k_p_* = 0.24. Thus, based on these and previous results from the literature, the dielectric, ferroelectric, and piezoelectric behavior, as well as structural and microstructural properties, of the studied samples depend not only on the composition, but also on processing.

## Figures and Tables

**Table 1:** Average, mode, and interquartile range data of grain size distribution obtained from analysis of SEM micrographs for the studied compositions.

Sample (mol % BKT)	Average Grain Size (µm)	SD	Mode (µm)	Interquartile Range (µm)
0	1.86	1.15	1.10	0.98
3.0	2.43	1.33	1.66	1.48
6.0	1.14	0.61	0.80	0.68
9.0	0.91	0.44	0.68	0.60
10.0	0.73	0.31	0.56	0.50
12.5	0.66	0.33	0.47	0.45
20.0	0.63	0.28	0.51	0.45
24.5	0.55	0.25	0.41	0.37

**Table 2:** Dielectric, ferroelectric, and piezoelectric properties of the BNT-BKT-BT prepared samples.

Sample (mol % BKT)	𝜀′	*tanδ*	*P_r_* (µC/cm^2^)	*E_c_* (kV/cm)	Q_mr_	*k_p_*	Q_me_	*k_t_*	*d*_33_ (pC/N)
0	621.1	0.04	33	51.7	143.0	0.15	35.2	0.45	72
3	617.8	0.04	27.5	49.5	141.7	0.16	31.1	0.43	73
6	711.8	0.03	26.9	40.6	144.8	0.19	43.4	0.41	79
9	858.0	0.06	25.1	36.9	113.5	0.18	18.1	0.41	66
10	1490.5	0.05	34.4	36.9	60.1	0.24	12.5	0.52	109
12.5	1505.8	0.06	29.4	27.2	40.0	0.24	10.2	0.50	126
20.0	1435.8	0.07	19.2	20.9	32.0	0.16	28.5	0.33	71
24.3	1327.4	0.07	18.2	15.9	29.4	0.16	28.9	0.32	70
